# Pedestrian behavior during evacuation from road tunnel in smoke condition—Empirical results

**DOI:** 10.1371/journal.pone.0201732

**Published:** 2018-08-29

**Authors:** Jakub Porzycki, Natalia Schmidt-Polończyk, Jarosław Wąs

**Affiliations:** 1 AGH University of Science and Technology, Faculty of Electrical Engineering, Automatics, Computer Science and Biomedical Engineering, al. Mickiewicza 30, 30-059 Kraków, Poland; 2 AGH University of Science and Technology, Faculty of Mining and Geoengineering, al. Mickiewicza 30, 30-059 Kraków, Poland; University of the West of England, UNITED KINGDOM

## Abstract

Five evacuation experiments were performed in a road tunnel in order to test how pedestrians react when exposed to reduced visibility, how the decision making process is carried out, and finally what is the impact of various circumstances like: different level of smokiness, competitive behavior or learning effect on an evacuation process. In four experiments pedestrians were exposed to artificial, non-toxic smoke. During evacuation of a group of people gathered in low and moderate level of smokiness (when *Cs* < 0.5*m*^−1^) we observed multi-line patterns created by pedestrians. Decision making was engaged in only by the first group of passengers, while under heavy smokiness *Cs* > 0.7*m*^−1^ we have observed decision making by small groups and characteristic double-lines patterns. In four experiments the same group of participants was involved, and a learning effect was observed: increasingly shorter pre-movement time and decreasing time required to leave the main tunnel. We show, that movement speed in smoke is influenced by the evacuees’ attitude and familiarity with environment and evacuation procedures and not only by the visibility level.

## 1 Introduction

In many countries, one can observe a growing tendency in the development of road tunnels. Disasters which occurred in tunnels show that safety issues are extremely important [1]. Among many technical aspects, the human factor, namely people’s response to a danger situation is one of crucial issues [2]. On the one hand, tunnels are a very useful element of road infrastructure, and on the other hand, one can point out some risks associated with the use of tunnels.

Accidents involving buses are very dangerous in tunnels: firstly, passengers have to leave the bus and secondly, they have to evacuate from the tunnel. Over the past few years, many such accidents have been reported, for instance fire of a bus in Taojiakuang Tunnel in Weihai, Shandong Province, China, on May 9, 2017 (12 fatalities), fire of a school bus in Jack Lynch Tunnel in Cork, Ireland, on Jun 4, 2013, accident in Dullin Tunnel, France, on Jan 18, 2004 or bus fire in Homer Tunnel, New Zeland, Nov 3, 2002 [3].

Our main aim is to investigate the human—infrastructure interaction. Human behaviors and interactions during dangerous situations should be taken into account by tunnel designers and people responsible for the safety of tunnel operation. According to our best knowledge, this is the first article considering a trial evacuation of a big group of people assembled in a bus in a road tunnel in smoky conditions. Namely, experimental results regarding the response of people exposed to considerably reduced visibility (caused by artificial smoke) in a road tunnel is taken into consideration. People have been gathered in a bus in order to reflect a scenario, where a large group of people are faced with a dangerous situation in a smoke filled road tunnel. This facilitates an investigation of the influence of grouping behavior on the evacuation process. In fact this is one of the worst case scenarios, when a ventilation system is not activated during a fire, and there is a large group of passengers gathered in one vehicle.

The main objectives of the performed experiments can be expressed as follows:

- response of pedestrians in reduced visibility, including reactions to different conditions,
- learning effect after repeated evacuation experiments,
- way-finding and decision making during evacuation in reduced visibility,
- relations between desired and actual velocity in smoke condition,
- comparison of influence of competitive vs. non-competitive behavior on evacuation process in reduced visibility conditions.

The article is organized as follows. Section 2 presents related works, while Section 3 includes the setup of experiments, tunnel parameters and experiments’ methodology. Results from experiments are presented in Section 4, we analyze: decision on starting evacuation, path selection, evacuees flow at selected checkpoints, movement speed and influence of smoke and initial position in bus. Finally, Section 5 includes discussion of results and concludes the whole research.

## 2 Related works

One can point out many significant articles related to human response to different evacuation conditions. It should be stressed that the behavioral aspects of an evacuation process have become an increasingly more important subject of research over the last years.

Behavior of people during an evacuation in a road tunnels in case of fire would be different than in regular buildings [4]. Tragic consequences of fires in tunnels [1] encouraged various researchers to conduct studies focusing on different aspects of an evacuation: [5–10] etc.

Motorists’ responses during an unannounced evacuation drill in a road tunnel were presented in [11]. A group of 29 participants believed that they took place in a driving behavior experiment, and inside the tunnel they noticed a blockade: a car and smoke. In such conditions: human—infrastructure interactions, observed behaviors and emotional states/reactions during a fire emergency were the subjects of research. The authors observed that decision–making process including delays in leaving cars and participants selecting emergency exits. Moreover, emotional states and other issues were assessed using questionnaires and interviews. Observed social influence and interactions between participants were a very important outcome from the research.

One of the most important decisions of an evacuee is choosing an appropriate exit. This has been investigated in a number of studies e.g. [12, 13]. Way-finding systems are a crucial factor that influences the evacuees’ decision on exit choice [9, 14, 15].

We have not found any articles dealing with trial evacuation of people from buses in a road tunnel in reduced visibility conditions. However, a research group from Murray State University and Auburn University considered different issues regarding evacuation from a school bus located in a road [16, 17]. They analyzed the evacuation process of different groups of children. They pointed out that “bus fire propagation tests indicate that the available time for successful evacuation is approximately *3–5 min*”. Interestingly, a group from Kanazawa University in Japan analyses results of different computer simulations of tunnel fire [18] and showed that if a bus is involved in a tunnel fire, the number of people requiring help leaving the tunnel tends to increase.

Some papers described the evacuation process and the behavior of evacuating people in rail tunnels and the metro: [14, 19–22]. Studies on movement speed and exit choice during evacuation of a rail tunnel were also described in [12]. In the research, a group of 100 participants (individual persons) was asked to leave a trail tunnel without any additional information about the layout of the tunnel and the available technical infrastructure. The tunnel was filled with artificial smoke with acetic acid as the irritant. The aim of the research was to assess the influence of infrastructure like emergency signs, lights etc. on the evacuation process.

Recently, experimental research on evacuation parameters in a smoke-filled road tunnel was presented in [23]. Participants, equipped with a flash light and a stopwatch among others, were asked to leave a smoke filled area in several experiments with different conditions respectively compared against a normal situation and evacuation. Next, the relations between the extinction coefficient and maximum, minimum and mean walking speed were examined. Finally, the authors compared their results with reference data from literature. Additionally, the effectiveness of guide lights was investigated for different extinction coefficients.

It should be stressed that simulation techniques and virtual reality methods can also be helpful in ensuring the safety of tunnels. For instance decision—making process during a tunnel evacuation in virtual reality was presented in [24]. Participants were asked to leave a tunnel using a joypad navigation implemented in a CAVE environment, a SketchUp 3D modelling software and Unity3D game engine. The results were used to calibrate a proposed floor field, cellular automaton—based model of evacuation.

Nowadays, many problems regarding evacuation are solved with numerical modeling. Literature demonstrates the utility of those tools for examining different aspects of evacuation in tunnels, i.e. the impact of smoke on pedestrian walking speeds during the evacuation [25], where simulations’ results are based on the experimental data obtained in real conditions: [26, 27]. One can also point out another interesting work [28], where different evacuation simulations (generated using Pathfinder and FDS+EVAC) were compared with data from [11]. The influence of different circumstances and parameters is discussed in the paper. Namely, the authors consider the influence of fear, information processing, unwillingness to abandon property and social influence on the evacuation process. Moreover, the impact of emergency exit signs on the evacuation process is described by [29]. However, the basic limitation of using numerical modeling is the necessity of validating different simulations against experimental results [22, 30, 31].

It should be stressed that carrying out research on evacuation in tunnels on a real scale is exceptionally difficult. It is related to some risks, it requires good organization and support of various units (i.e. emergency services). Unfortunately, many scenarios are impossible to check due to safety or logistical reasons.

## 3 Experiments

### 3.1 Tunnel and participants

**Fig 1:**
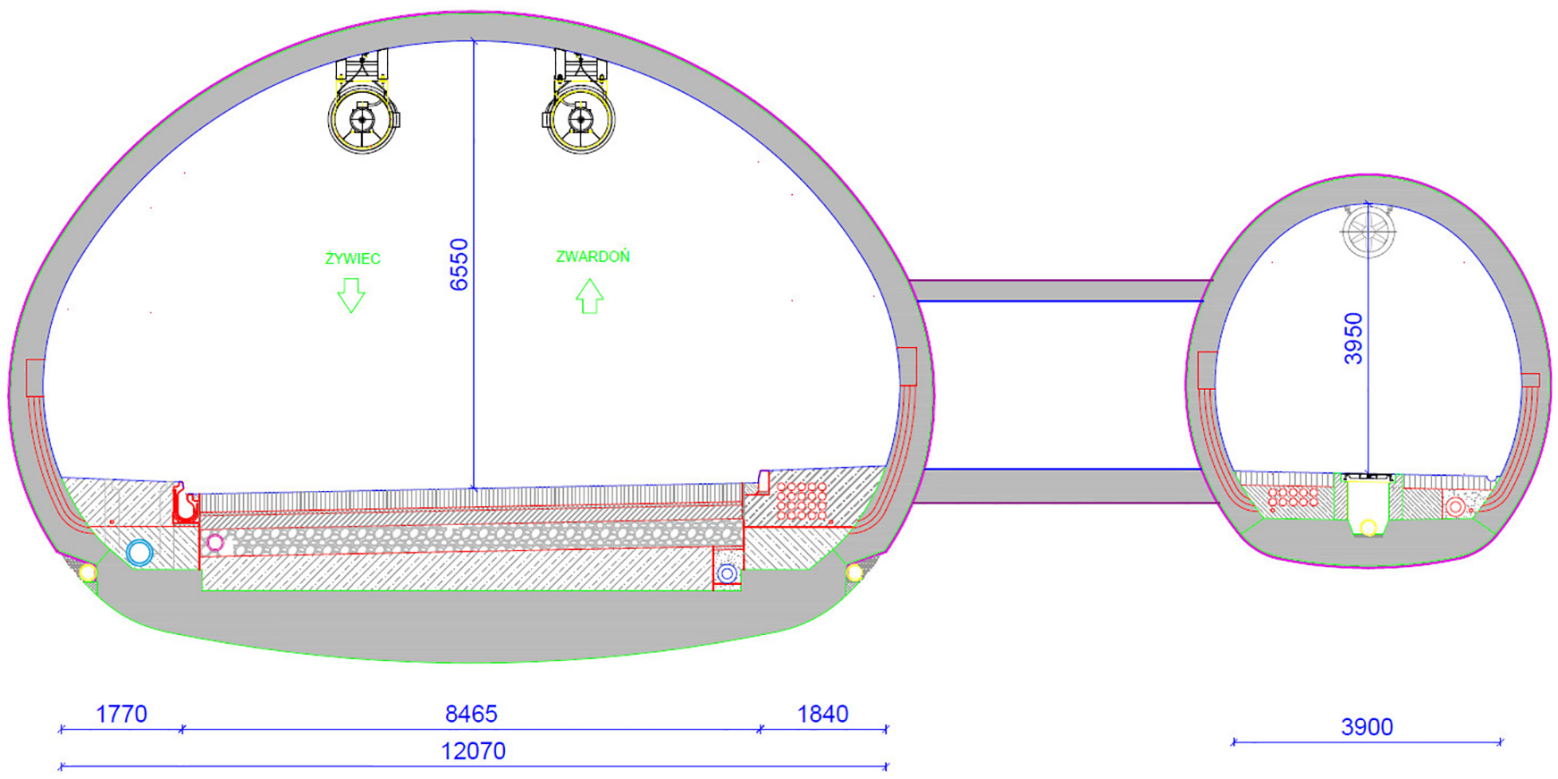
“Emilia” tunnel cross-section, all the dimensions are specified in [*mm*].

Experiments have been carried out in “Emilia”, a 678 m long road tunnel located in south-west Poland. The tunnel compromises two parallel tubes: one tube of a bidirectional road tunnel and an evacuation tube (see Fig 1). The main tunnel tube comprises two traffic lanes, one in each direction. The tunnel axis has a constant longitudinal slope of 4.0% directed towards the southern portal of the tunnel. There are four cross passages connecting the road tunnel with the evacuation tunnel. A detailed description of the tunnel is provided in appendix (S1 Appendix).

The entire research has been divided into two parts. The first part—evacuation without smoke—was carried out on April 24, 2016. The second part—four evacuation experiments with artificial, non-toxic smoke—was carried out on October 19, 2016. Groups of participants in the first part (a reconnaissance) and the second part were different. In the first part, one evacuation experiment (experiment 0—Table 1) was performed, while in the second part there were four evacuation experiments (experiments 1–4—Table 1). For experiment 0, we invited a group of participants consisting of 28 tunnel technology professionals, while for the second part (experiments 1–4) we invited a group of 50 students.

**Table 1:** Short characteristics of all performed experiments including estimated visibility, group familiarity with tunnel and tasks for participants.

Number	Level of smokiness	Tunnel familiarity	Task for pedestrians
Experiment 0	No smoke	Yes	To evacuate
Experiment 1	Light smoke ∼ 0.1 − 0.2*m*^−1^	No	No direct, expressed task
Experiment 2	Moderate smoke ∼ 0.4 − 0.5*m*^−1^	Yes	To evacuate
Experiment 3	Heavy smoke ∼ 0.8 − 0.9*m*^−1^	Yes	To achieve possibly the best time
Experiment 4	Heavy smoke ∼ 1.0 − 1.1*m*^−1^	Yes (different starting point)	To evacuate

Participants of experiments 1-4 were asked about their familiarity with the conditions that occur during experiments. Only 2 of them have ever participated in a real or trial tunnel evacuation, and only 5 have ever tried to move/evacuate in smoke conditions. On the other hand, 41 participants took part in a real or trial evacuation in the past.

### 3.2 Experiments assumptions and scenario

**Fig 2:**
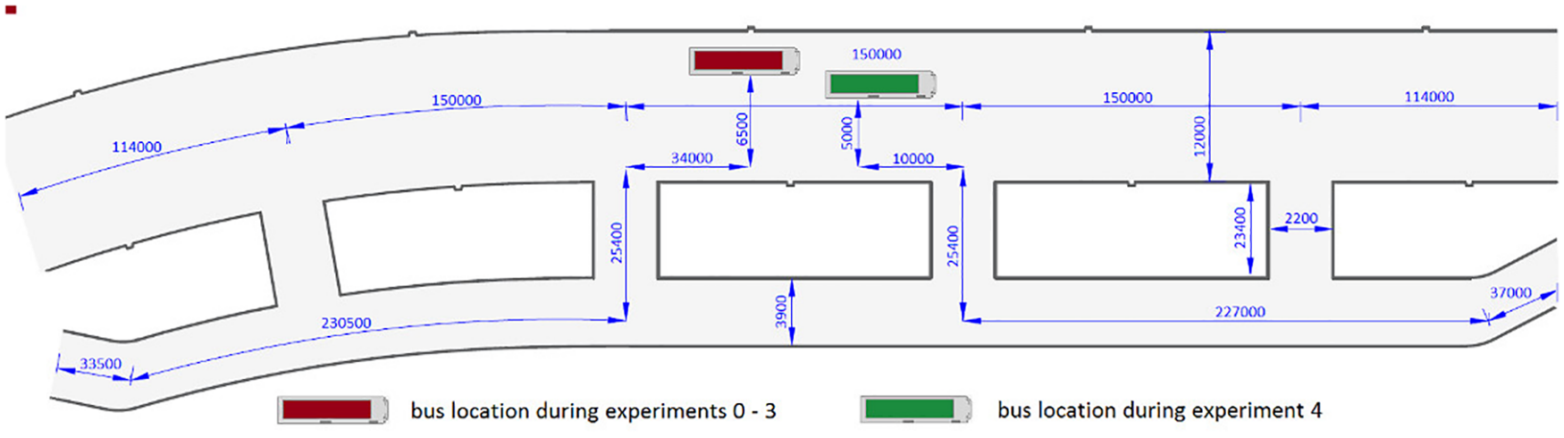
Experiment configuration. Bus with participants were stopped in the left lane, in the middle between the second and third evacuation tunnel entrance. Smoke generators were located in front of the bus. Please note, that in order to improve readability of this figure we use different vertical and horizontal scaling. Due to the same reasons, the size of the bus is out of scale. All dimensions are listed in millimeters [mm].

The main assumptions of the experiments are presented in Table 1. In experiment 0, participants were asked to evacuate from the tunnel. In experiment 1, the participants did receive any instructions as to the purpose of the experiment, suggested behavior in case of smoke, tunnel infrastructure or bus position in the tunnel. The bus was driven into the tunnel without stopping, in order to ensure an element of surprise for participants. In this experiment the bus stopped in the middle of the tunnel, just like in experiments 2 and 3. Detailed experiment setup is presented in Fig 2.

The signs for the participants to start evacuating are similar to these which occur in a real accident including: presence of smoke, activation of the emergency procedures in the tunnel such as voice communications and emergency lights. Note that there was no special start signal from the organizers and the bus driver was instructed to stay in his place and not to give any prompts to participants, except the opening middle doors, when he heard the voice alarm.

The following experiments were carried out in almost the same manner with some variations. In experiment 2, students received instructions as to how to behave and were given safety rules in the event of a fire, which can be seen in appendix A. In experiment 3 participants were challenged to evacuate as quickly as possible. Finally, in experiment 4, the bus stopping place moved to a different position in the tunnel, as shown in Fig 2. Moreover, while driving into the tunnel participants had their eyes closed. In this experiment, both doors on the bus were opened, whereas in experiments 1-3 only the middle door was opened.

**Fig 3:**
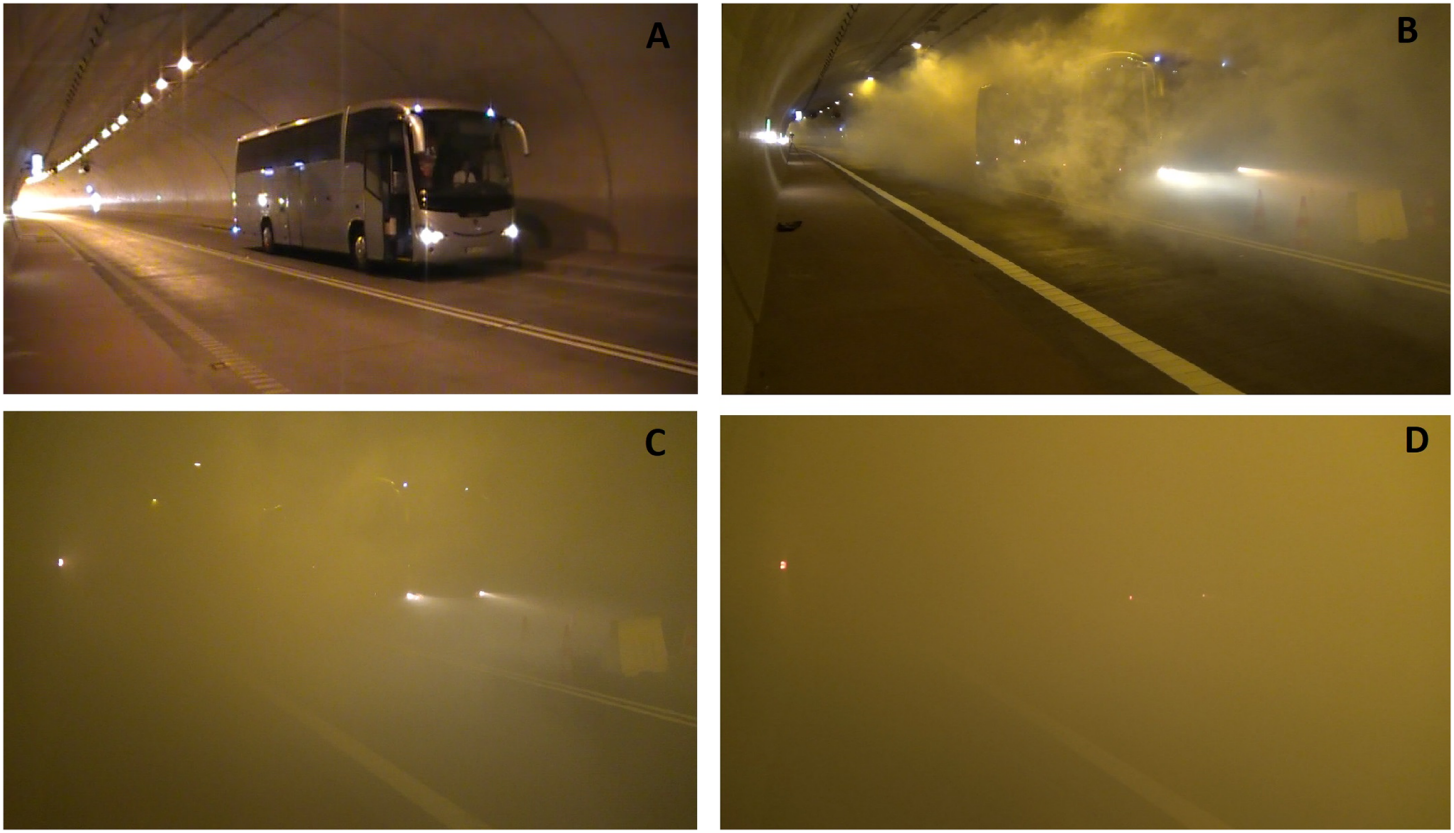
Comparison of representative visibility level in experiments 0-3, consecutively at Fig 3 A-D.

The level of smokiness increased in consecutive experiments, as shown in Fig 3. Our aim was to test the worst case scenario, when the ventilation system (namely a set of jet fans) is not activated. Application of a ventilation system causes removal of smoke from the tunnel and it would not be possible to investigate the impact of smoke on the evacuation process. Thus, the ventilation system was only used to remove the artificial smoke after each experiment. Evacuees’ flow during each experiment was measured using a set of video and infrared cameras.

Directly after experiments 1–4, we asked the participants to complete a survey about their observations and impressions during the evacuation. Participants were also encouraged, to write down their observations and impressions from each experiment.

### 3.3 Methodology—Summary

We invited two groups of participants to attend experiments—emergency evacuation in a road tunnel from a bus: without smoke, experiment 0 (group 1), in smoky conditions, experiments 1-4 (group 2). It should be stressed that participation in the experiments was voluntary and all participants signed a written consent for their voluntary participation in the experiment. During all experiments, all safety procedures were applied, and in particular the participants were under constant supervision of the fire brigade, medical emergency services and the organizers. The course of experiments was agreed with institutions responsible for security: the fire brigade, police and medical emergency services. We also obtained a statement from the Bioethical Commission at the District Medical Chamber in Krakow, to the effect that the need for ethical approval is waived for the experiments.

During all experiments we manipulated one main variable, namely the visibility level (amount of smoke was increasingly greater in consecutive experiments). Additionally, during experiment 3 we asked participants to obtain *the best individual evacuation time*, and as a consequence, competitive evacuation was observed. During the last experiment we changed bus stopping place, in order to impede the localization task for participants, and simultaneously two doors of the bus were open during evacuation.

Regarding the methodological point of view, we applied a direct observational method, namely structured observation, which is located between a naturalistic observation and lab experiments. The whole staff were neutral during experiments: the bus driver was instructed not to suggest any solutions to participants, firefighters securing the safety of the participants were hidden and did not participate directly in the experiment.

## 4 Results

### 4.1 The decision to start the evacuation

We analyze the decision making process of individuals on starting the evacuation in experiment 1, since this is the only experiment when participants unfamiliar with the tunnel encountered a new, emergency situation (see Table 1). Before entering the tunnel participants knew only that they will take part in an evacuation experiment in the tunnel, they were not informed about experiment details and did not expect artificial smoke. From the participants’ point of view, the sequence of events was as follows:

- bus enters the tunnel and stops in the middle,
- then smoke begins to surround the bus,
- after a moment a siren starts to sound,
- voice alarm messages in Polish and English are initialized: (*Attention please, attention please. Fire alarm. Leave your car and go to the nearest emergency exit*).

After the bus had stopped, participants occupied their seats. Their behaviors are the same when they notice smoke, they do not take any actions except talking about this situation, however some of them start to become alarmed and somehow nervous. When the siren starts to sound (*t* = 13*s*) all of them become alarmed, but still remain sitting in their seats.

A voice alarm message was needed to encourage the first evacuees to start evacuation (*t* = 27*s*). Two groups of four and five persons decided to leave the bus at this stage. It takes another 23 seconds (*t* = 50*s*) until the next few persons get up from the seats and leave.

This observation is another confirmation, that in case of an accident, people usually tend to maintain their initial roles (e.g. as passengers), as long as they don’t get a clear message telling them what to do. Similar behavior was observed in many underground fires i.a. the Kings Cross fire [2], the Zürich Metro fire [32] or the Mont Blanc tunnel fire [1]. In our experiment even an alarm siren by itself was not enough to convince participants to start the evacuation. Clear instruction was needed to initiate evacuation. Moreover, a voice message triggered only a small group to start evacuation, the remaining evacuees follow them 23 seconds later. Similar indifference to a siren alarm was observed in a trial evacuation of the Stockholm metro [32, 33].

It is important to stress the role of leaders, since they are usually the triggers that start and direct the evacuation of a particular group [34]. In such a case, a bus driver is a natural leader, but in this case he was instructed to remain passive and inactive. Thus, we can observe that some participants spontaneously adopt this role—encouraging others to move and pointing out the proposed evacuation path (see section 4.2). Nevertheless, passive behavior of a natural leader slows down the evacuation, until a decision has been made by other participants. On the basis of conducted experiments, it is difficult to determine how evacuees’ reaction time changes in case of the absence of a natural leader. However, the fact that participants start evacuation after hearing the voice message suggests that clear instructions are an important trigger to evacuate.

Finally, during experiment 1 we observed the longest pre-movement time (compare evacuees flow in section 4.3). In this case, the first person left the bus after 37 seconds, while for experiment 0 it was 11 s, and 3-6 s for experiments 2-4 s. It clearly shows the influence of the learning effect on pre-movement time. Previous experiences and familiarity with evacuation procedures allow participants to react faster and to reduce the time required to abandon a dangerous area.

This was confirmed by participants in post-experiment survey. Their comments show the importance of experience in such a situation. After the first experiment, few observations were noted which mention *indecisiveness* and *tardiness in starting evacuation*. On the other hand, after second experiment we obtained 7 comments that show the learning effect on the decision to start evacuating and choose an escape path. Interestingly, “Fire Evacuation Instruction” (see Appendix) given to participants before experiment 2 was mentioned only four times—mostly in terms of bending during evacuation in smoke.

### 4.2 Path selection

In terms of path selection we focused on experiments 1 and 4. In experiment 1, participants were not informed what will happen, thus this experiment should be most similar and true to actual incidents in tunnels. In experiment 4, the bus stopped in a different place—participants do not know where in tunnel they are (blindfolded eyes). Therefore, in this experiment, we can analyze the path selection process in difficult conditions with the lowest visibility.

**Fig 4:**
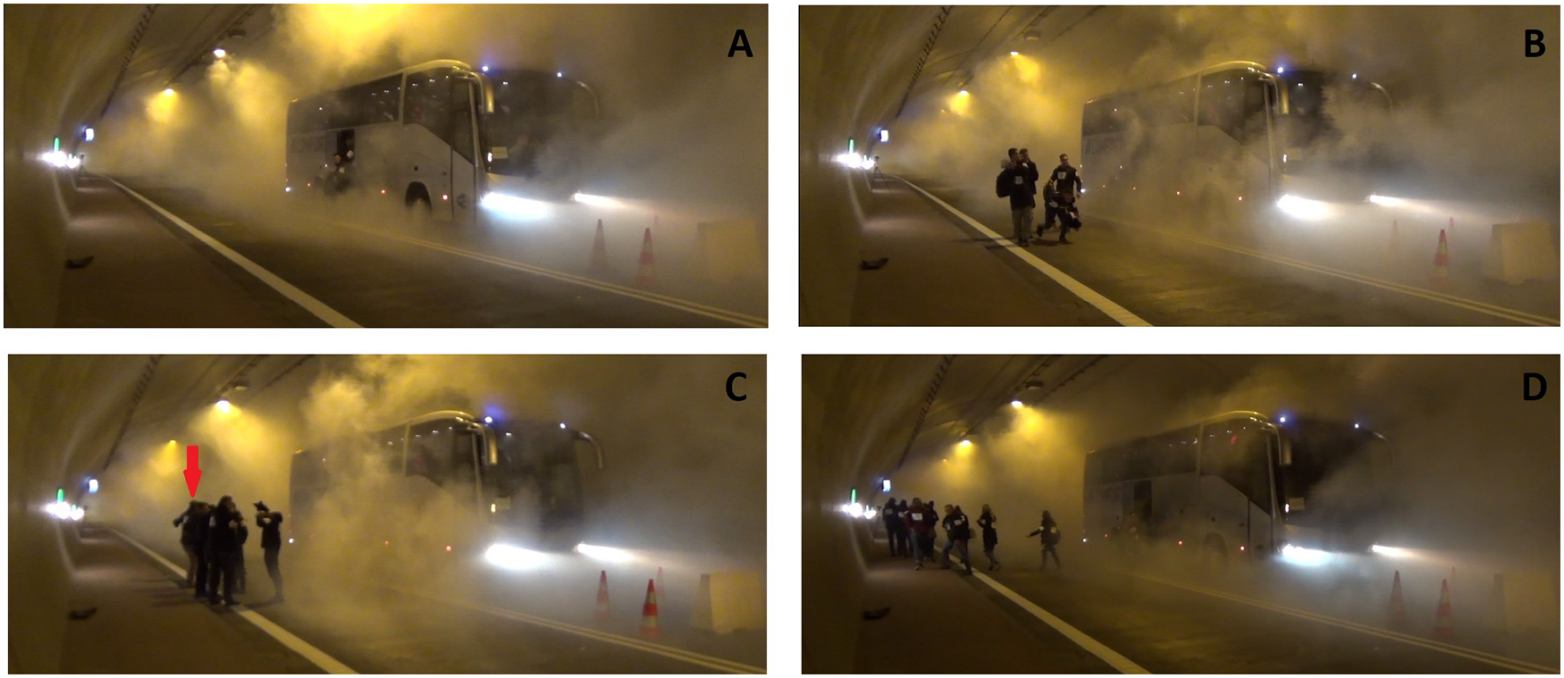
Consecutive stages of path selection by participants during experiment 1. Fig 4 A: The first person is leaving the bus (*t* = 37*s*). Fig 4 B: The group of first four evacuees is looking around and discussing (*t* = 44*s*). Fig 4 C: Path selection. A student can be seen on the left pointing the path of evacuation (*t* = 55*s*). Fig 4 D: Latter stage of evacuation, subsequent persons leaving the bus follow the rest of participants (*t* = 71*s*).

During experiment 1 pre-movement time (this paper focuses on tunnel evacuation, thus we assume that time of evacuees’ movement inside the bus is part of pre-movement time) was large, the first person leaves the bus 37 seconds after initialization of the alarm system (*t* = 37*s*), see Fig 4a. Process of path selection during experiment 1 is presented in Fig 4. At the beginning, only four persons decided to leave the bus. Firstly, they did not know what to do, the participants just stayed in a group and tried to look around (Fig 4b). The decision on path selection (*t* = 55*s*) was taken in discussion with a second group of five persons, when participants decided to follow the evacuation signs. In Fig 4c the moment when the decision was made was captured, a student can be seen on the left (indicated with an arrow), pointing out the suggested direction of motion.

It is worth noting, that *after selection of an evacuation path, all remaining participants followed*, without stopping (and discussing), as presented in Fig 4d. When a subsequent evacuee leaves and notices a group of participants going in some direction he/she follows them without hesitation. After the first two groups (9 persons) we observe only 2-3 cases when evacuees read the evacuation sign, however they never stop and always follow the group. This is another example of herding behavior during evacuation.

**Fig 5:**
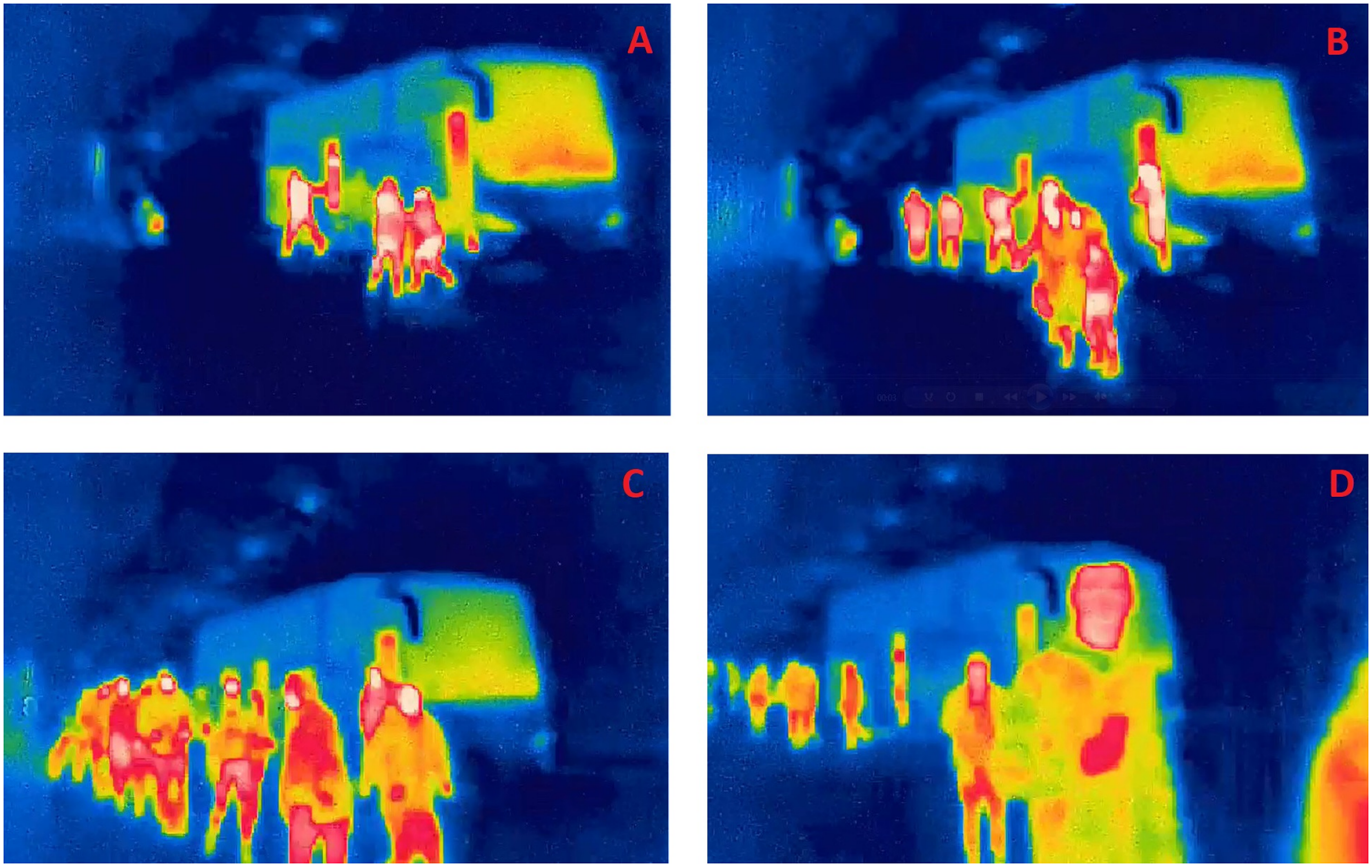
Consecutive stages of path selection by participants during experiment 4. Views from a thermal camera. Fig 5 A: First participants leave the bus (*t* = 8*s*). A collision occurs between participants in front of the bus. Fig 5 B: Different evacuation path selection between small groups from front and rear door at the beginning (*t* = 10*s*). Fig 5 C: In a latter part of experiment, most of the evacuees form an organized group, and head together to the same exit. (*t* = 24*s*). Fig 5 D: When last persons leave the bus, the evacuees outflow from bus decreased (*t* = 41*s*), as a result groups of pedestrians again choose different evacuation routes.

In experiment 4 we changed the bus stopping position in the tunnel and participants had their eyes blindfolded, thus they could not use the evacuation path they learned from previous experiences. Moreover, the largest amount of smoke was used in this experiment (see Table 1), thus observation of participants’ behavior was possible only with infrared camera (Fig 5). In contrast to the previous experiments, during experiment 4 both bus doors were open.

Participants began the evacuation without delay, the first person left the bus at *t* = 6*s*. Due to insufficient visibility, collisions between evacuees were observed (Fig 5a). At the beginning, participants leaving the bus through the front and rear door did not see each other, and as a result these two groups did not merge during the first stage of the evacuation (Fig 5b). Participants from the front door went to exit 3—the closer one, while participants that left the bus through the rear door went to exit 2—the same they evacuated in previous experiments. However, after few seconds, when more persons left the bus, they were able to form an organized group—where each person sees its predecessor, and follow the same direction (Fig 5c). The self-organized structure they formed—*double-line pattern* usually appears in high densities during two directional motion [35]. The majority of participants evacuated in this way. Finally, at the end of the experiment, when the density in front of the bus doors decreased, groups from the front and rear doors split up again (Fig 5d).

### 4.3 Evacuees flow

**Fig 6:**
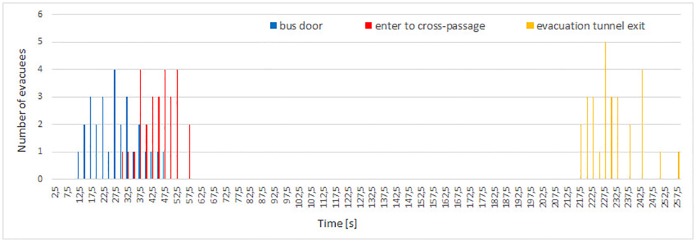
Flow of evacuees during experiment 0. The chart illustrates the appearance time of evacuees at consecutive checkpoints: bus door, entry to cross-passage and exit from evacuation tunnel.

**Fig 7:**
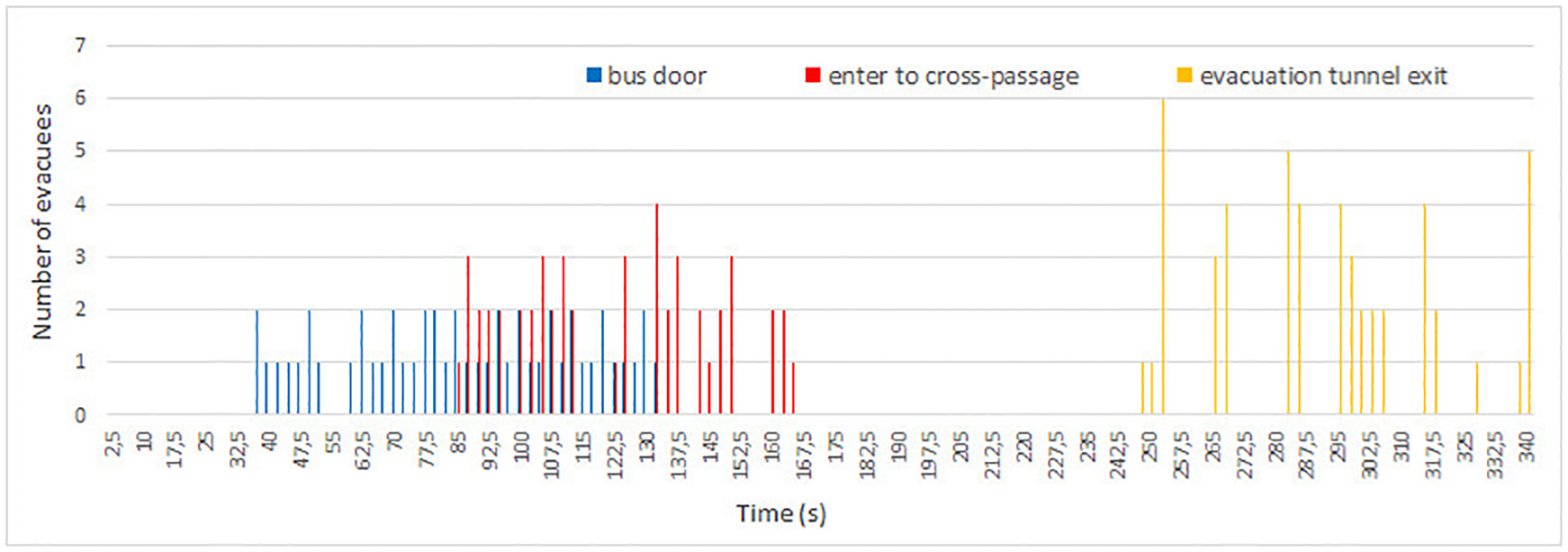
Flow of evacuees during experiment 1. The chart illustrates the appearance time of evacuees at consecutive checkpoints: bus door, entry to cross-passage and exit from evacuation tunnel.

**Fig 8:**
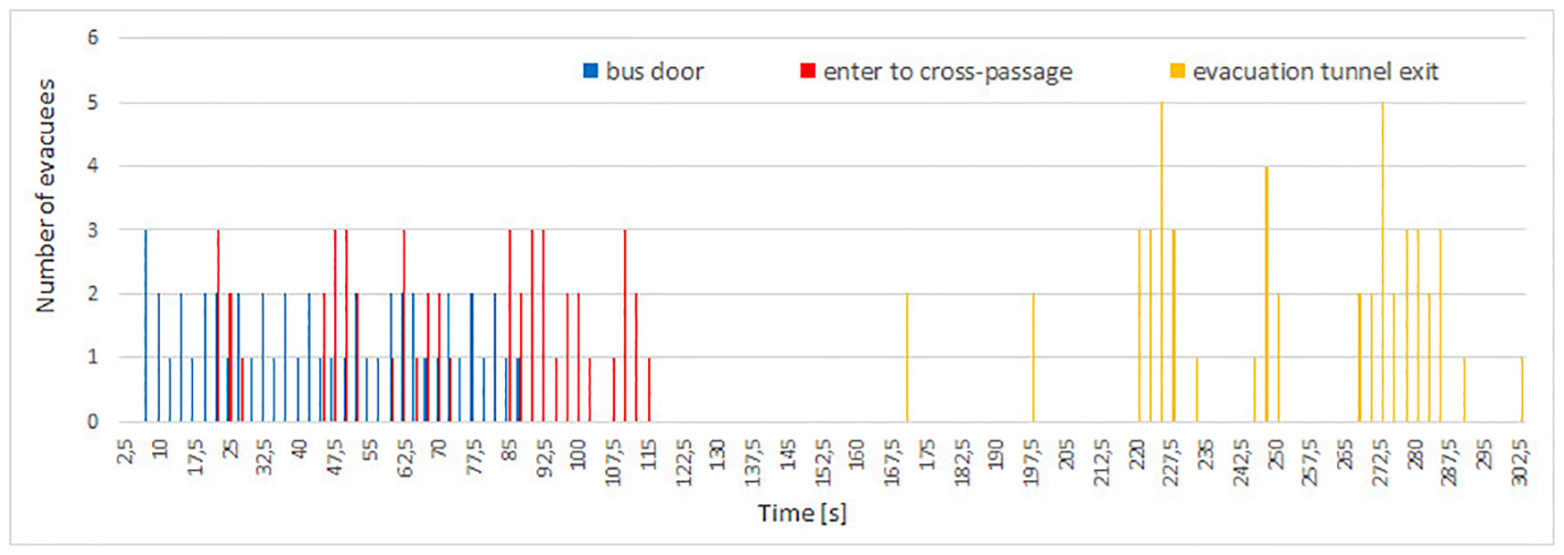
Flow of evacuees during experiment 2. The chart illustrates the appearance time of evacuees at consecutive checkpoints: bus door, entry to cross-passage and exit from evacuation tunnel.

**Fig 9:**
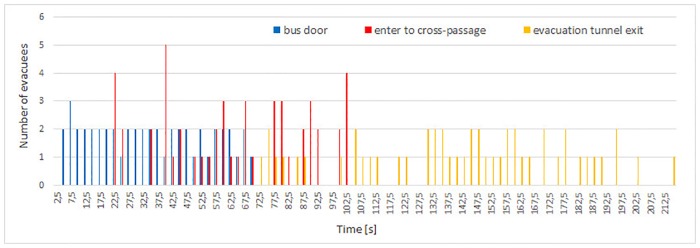
Flow of evacuees during experiment 3. The chart illustrates the appearance time of evacuees at consecutive checkpoints: bus door, entry to cross-passage and exit from evacuation tunnel.

**Fig 10:**
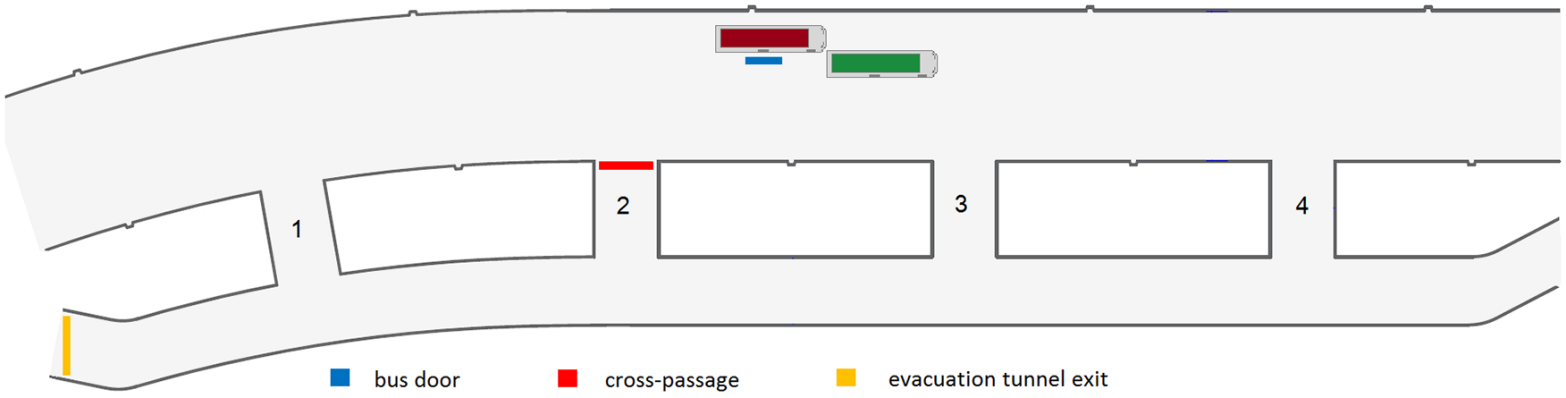
Location of checkpoints (bus door, cross-passage and exit) where evacuees’ flow was measured in experiments 0-3. Figure also shows the numbers of cross-passages between the main and the evacuation tunnel.

Evacuees’ flows for consecutive experiments 0-3 (due to different starting conditions, we excluded experiment 4 from sections 4.3, 4.4 and 4.5) are presented in Figs 6, 7, 8 and 9. The flow was measured at three checkpoints (see Fig 10), namely: at the **bus door**, at entrance to the **cross-passage** between the main tunnel and the evacuation tunnel and at the **exit** from the evacuation tunnel. Due to smoke, different setting of each camera and variable conditions during experiments we used manual calculation of flow. For the purposes of this analysis, data are discretized in time domain, with steps of 2.5 seconds.

Total evacuation times are respectively 254, 340, 301 and 215 seconds for consecutive experiments 0-3. The longest evacuation time occurred in experiment 1, while the shortest was measured in experiment 3. In case of experiments 0 and 2 the evacuation time was similar—the longer time for experiment 2 is due to higher number of participants—however the first 28 evacuees in experiment 2 left the tunnel after 254 s.

During experiment 0, evacuees flow through consecutive checkpoints is similar (see Fig 6). The structure of groups and order of evacuation has been roughly maintained. In this experiment two bus doors were opened, thus instantaneous flow through bus door achieves higher values (flow through bus door in experiment 0 lasts approximately 35 s. Interestingly, despite only one door open in experiment 3, in the same time span, 28 persons also managed to leave the bus, but instantaneous flow does not attain such high values).

On the other hand, in experiments 1 and 2, pedestrians formed groups during the evacuation process, which is clearly visible as a changing pattern on flow plots (Figs 7 and 8). For both experiments, flow of pedestrians measured at the bus door is low and constant. Later in the cross-passage one can observe higher maximal flow, and longer time gaps between groups of evacuees. Finally, in the exit from evacuation tunnel, instantaneous flow reached the highest values and the time gaps between plot bars are the biggest. This indicated existence of well formed groups of pedestrians. The described process is slightly more visible during experiment 2.

In experiment 3 formation of large group was observed only in the cross-passage (see Fig 9). Between the bus doors and the evacuation tunnel pedestrians moved in dense smoke. Therefore, in order to help themselves to find the evacuation exit and to prevent collisions, evacuees have formed small groups and later double line patterns similar to these shown in Fig 5b and 5c. This was the reason for high and sparse bars in the flow plot for cross-passage. After entering the evacuation tunnel visibility improved significantly, and evacuees started to rush towards the exit, which finally caused a steady, but low flow at the exit checkpoint.

In experiment 1 one can observe the highest time span between leaving the bus and entering the evacuation tunnel—48 seconds (see Fig 7), while in other experiments this time span varies between 16 and 18 seconds (Figs 6, 8 and 9). Thus, in experiment 1, the time of leaving the main tunnel is the highest, from 84 to 163 second after the siren starts, in experiment 0 it was 32 to 62 second (evacuation time from the bus and the main tunnel for experiment 0 cannot be compared with other experiments due to a smaller number of participants and higher number of bus doors (2) opened). During both experiments 2 and 3, the first person appeared in the cross-passage at *t* = 21*s* and the last at *t* = 114*s* and *t* = 102*s*, respectively.

For experiments 2 and 3, evacuees flow at bus door and passage checkpoints are similar (see Figs 8 and 9). However, pedestrians reached the exit from the evacuation tunnel in experiment 3 much faster than in experiment 2. It shows that pedestrians motivation influences mostly movement in the evacuation tunnel, while time of leaving the most dangerous area—the main tunnel, is mostly determined (besides smokiness level) by evacuees’ familiarity with the situation, scenario and layout of the tunnel.

### 4.4 Movement speed

We calculated movement speed for experiments 1-3. Movement speed in the main tunnel and the evacuation tunnel was calculated separately, since the differences in conditions in these tunnels are significant—there is no smoke in the evacuation tunnel. Due to the size of the tunnel and the heavy smoke, determination of exact trajectories (and exact speed respectively) would be extremely hard. Thus, we calculated evacuees’ speed in a simplified way, as the distance to travel divided by walking time. Fridolf et al. propose to call this method *modeling speed* [12]. However, this method is widely used in similar experiments [4, 31]. The calculated movement speeds for the main and the evacuation tunnel in consecutive experiments are shown in Table 2.

**Table 2:** Movement speed for the main and the evacuation tunnel for experiments 1-3. First 9 persons, who stopped and discussed after leaving the bus during experiment 1 were excluded from the statistics.

Experiment section	Minimum	Maximum	Mean	Std. deviation
experiment 1 the main tunnel	0.895	1.211	1.056	0.083
experiment 1 the evacuation tunnel	1.542	1.808	1.706	0.058
experiment 2 the main tunnel	0.917	2.422	1.321	0.375
experiment 2 the evacuation tunnel	1.489	1.953	1.635	0.081
experiment 3 the main tunnel	0.893	2.044	1.221	0.295
experiment 3 the evacuation tunnel	2.569	5.760	3.835	0.719

Movement speed in the evacuation tunnel (Table 2) depends only on evacuees’ attitude. During experiments 1 and 2 all participants move with almost the same speed—mean: 1.706 and 1.635 $\frac{m}{s}$ with a standard deviation of 0.058 and 0.081, respectively. While in experiment 3, they were instructed to achieve possible the best individual times, and they have moved much faster. The mean speed was equal 3.835 $\frac{m}{s}$ (std dev. 0.719) with a maximal speed of 5.760 $\frac{m}{s}$. This difference clearly shows how motivated the evacuees were in experiment 3.

In the main tunnel (Table 2) only minimal speed remains the same for each experiment 1-3 (0.893—0.917 $\frac{m}{s}$). The mean speed was the highest during experiment 2 (1.321 $\frac{m}{s}$), slightly less in experiment 3 (1.221 $\frac{m}{s}$) and the lowest in experiment 1—only (1.056 $\frac{m}{s}$). For maximal speed, the order is the same—the highest during experiment 2 (2.422 $\frac{m}{s}$), 2.044 for experiment 3 $\frac{m}{s}$ and only 1.211 $\frac{m}{s}$ for experiment 1.

Our values of average speed in smoky conditions are lower, than values reported by Seike [4] et al., for instance 1.22 *m*/*s* (*c*_*s*_ = 0.8 − 0.9) vs. 1.79 *m*/*s* (*c*_*s*_ = 0.83). It can be explained by the grouping behavior effect in our experiments, when the speed of a group is lower than the speed of particular pedestrians [35].

The obtained results clearly show that movement speed in smoke is not only depended on smoke density (visibility—extinction coefficient), but is also influenced by pedestrians’ attitude and familiarity with the tunnel. In experiments 2 and 3 we used more smoke, thus visibility was lower (see Table 1), but evacuees achieved higher speeds. In previous research [4, 12, 31] pedestrian movement speed is usually considered as a function of extinction coefficient only. The described results suggest that both familiarity with the environment (experiment 2) and attitude (experiment 3) influence pedestrian movement speed as well.

Such a fact suggests that the negative influence of reduced visibility on total evacuation time can be compensated by evacuees’ motivation and learning effect. It confirms recent results by Seike et al. [23]. One should remember that due to safety reasons, in this experiment we used non-toxic, non-irritant smoke, so all effects related with pedestrians’ intoxication were omitted for obvious reasons. In our experiment, smoke (reduced visibility), did not decrease the total evacuation time in significant way, however it can affect individual pedestrians. According to our observations smoke affects evacuees in number of ways, namely: leads to collisions between evacuees (see Fig 5a), increases the possibility of stumbling (risk of stumbling was mentioned in participants observations), leads to separation of groups (see Fig 5b), brings the feeling of fear on uncertainty (see section: 4.7).

### 4.5 Movement speed and desired speed

In the context of pedestrian dynamics modeling it is worthwhile to analyze this case as a slowdown between desired and the actual speed in smoke. Desired walking speed is used in microscopic crowd dynamics models and it represents a maximal speed that particular simulated pedestrian want to achieve. This is a basis for the most popular types of pedestrian dynamics models, namely: Social Force Models [36] and Cellular Automata based models [37, 38].

Since there was no smoke in the evacuation tunnel and no obstacles were placed, the tunnel’s width was enough to enable high pedestrian speeds and easy overtaking of slower pedestrians. Thus, velocities characteristic to free movement were observed, however the small inclination (4.5%) slightly slowed down participants [12]. Finally, we assume that evacuee achieved their desired speed in the evacuation tunnel. For each evacuee we calculate their speed in the main tunnel as a percentage of desired speed (understood as speed of free movement in evacuation tunnel). This makes it possible to analyze how different conditions in experiments 1, 2 and 3 influenced pedestrians speed. Results of this analysis are presented in Table 3.

**Table 3:** Movement speed in main tunnel as a percentage of the desired speed (movement speed in the evacuation tunnel). In experiment 2, the first six persons decided to run in the main tunnel and then walk in the evacuation tunnel, this allows them to achieve a higher speed in the main tunnel. We consider it as a different desired speed in different part of the tunnel, and we present two versions of experiment 2 analysis (with and without “runners”). Similarly to the assumptions in section 4.4, in results for experiment 1 we excluded the first 9 persons, who after leaving the bus, stop and discus which evacuation path to select (see.: 4.2).

Experiment section	Minimum	Maximum	Mean	Std. deviation
experiment 1	51.15%	78.56%	62.56%	6.12%
experiment 2 all evacuees	57.83%	162.60%	80.73%	22.23%
experiment 2 without runners	57.83%	81.96%	73.17%	5.90%
experiment 3	26.44%	39.64%	31.46%	3.12%

In experiment 1 the mean speed of participants in the main tunnel equals to 62.56% of their desired speed. Surprisingly, in experiment 2 despite less visibility, evacuees were able to achieve 73.17% of their desired speed. Substantially smaller values are obtained with heavy smoke in experiment 3. Evacuees were able to achieve only 31.46% of their desired speed. One should note the low dispersion (spread) of results, standard deviation equals to: 6.12%, 5.90% and 3.12%, respectively, and the differences between boundary values (*max* − *min*) are 27.41%, 24.13% and 13,20%.

**Fig 11:**
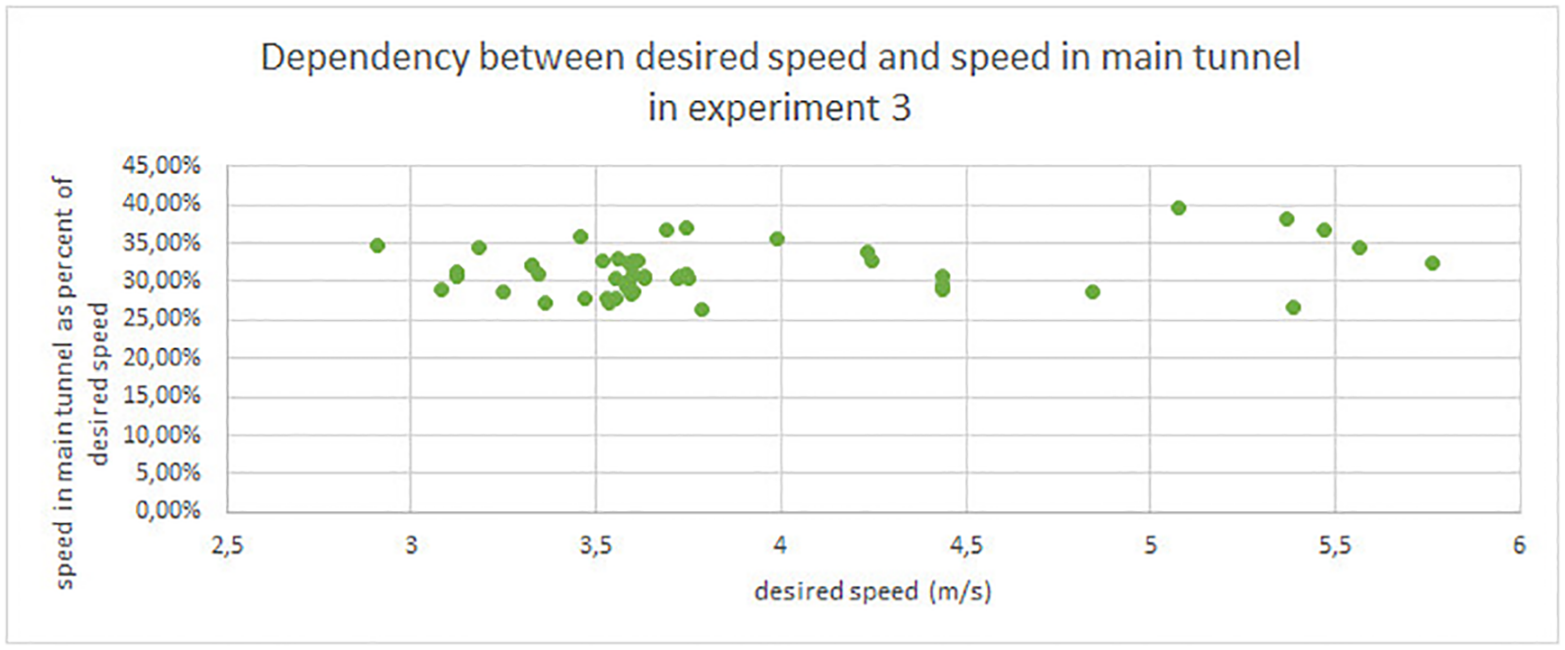
Dependency between the desired speed and the speed in the main tunnel in experiment 3.

Since the spread of the desired speed in experiment 3 is quite high (see Table 2)—$min=2.569\frac{m}{s}$, $max=5.760\frac{m}{s}$, $\sigma =0.719\frac{m}{s}$, using this data we analyze the dependency between the speed in smoke and the desired speed. Fig 11 illustrates how many percent of desired speed were remained during movement in the main tunnel. There is only weak, positive correlation between this data on account of the Pearson Correlation Coefficient = 0.2852, *p* = 0.046997 (*p* < 0.05). This suggests that in the considered conditions, a percentage speed decrease due to smoke is similar for all desired velocities (from range $2.5-5.7\frac{m}{s}$).

On the other hand, due to security and organizational reasons the number of examined scenarios as well as the number of participants in each scenario is limited. Therefore, the results described in this (sec. 4.5) and previous subsection (sec. 4.4) can be disturbed by the small size of samples. In order to address this issue in this paper, we focused only on the clearest dependencies. Nevertheless, further research is required to investigate the dependency between movement speed, and the desired speed in different smoke density.

### 4.6 Influence of initial position in bus on total evacuation time

**Fig 12:**
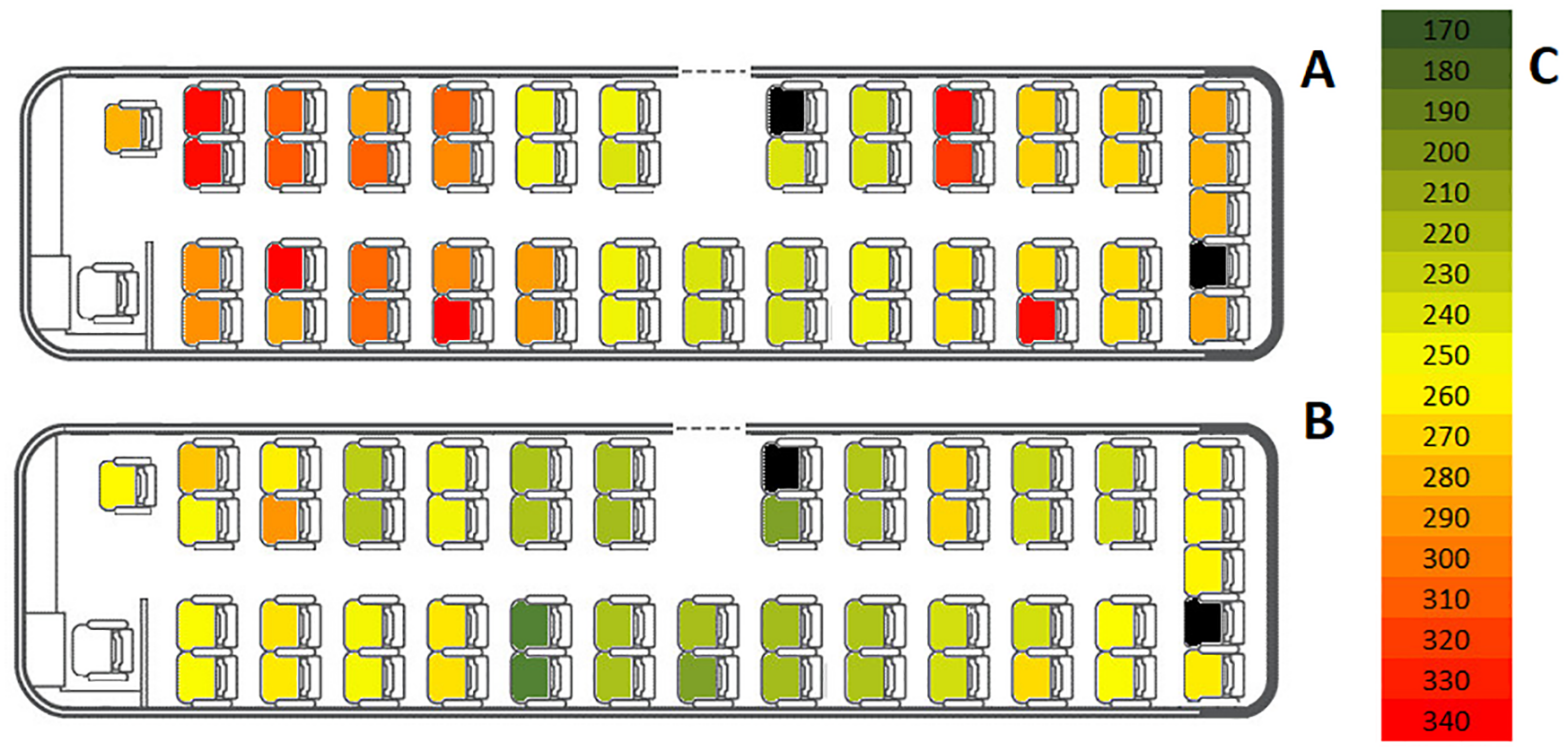
Dependency between position in the bus and evacuation order at exit from evacuation tunnel checkpoint in experiment 1 (Fig 12 A) and experiment 2 (Fig 12 B). The greener the seat, the faster this particular evacuee left the evacuation tunnel. Color scale (Fig 12 C) on both images is the same. Black spots indicates empty seats.

We analyze how initial position in a bus influenced total evacuation time (*hot seat* analysis). The overall dependence is presented in Fig 12. Results for experiment 1 are presented in Fig 12A, while for experiment 2 in Fig 12B.

The color scale used in both images is the same, it varies from 170*s* (dark green) to 340*s* (red). Thus, it is easy to observe the learning effect as the differences in evacuation time between experiment 1 and 2. Namely, every person evacuates faster in experiment 2.

One can easily notice clear, positive dependence between distance to the middle bus door and evacuation time. Another interesting dependence is the fact, that often persons who sit next to each other have similar evacuation times. This suggest that evacuees forms a stable dyads during the whole evacuation.

### 4.7 Influence of smoke on individual behavior

**Fig 13:**
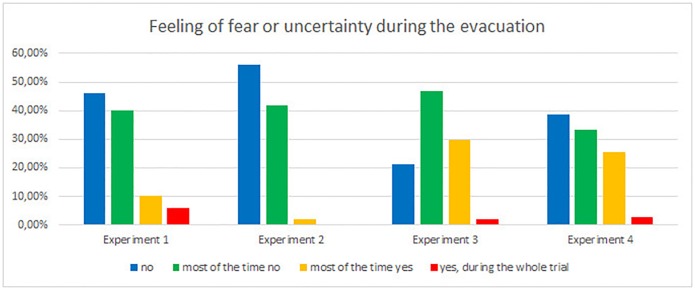
Feeling of fear or uncertainty among evacuees during consecutive experiments.

In the survey after each experiment, among others the evacuees were asked to state if they felt any fear or uncertainty during this experiment. The results are presented in Fig 13. During experiment 2, when smokiness level was moderate and the participants had knowledge of what will happen, evacuees felt the safest and most confided—98% chose *no* or *most of the time no* in the question about feeling fear. It is worth noting, that participants felt more confident during experiment 1 when they unexpectedly found themselves in an alarm drill, than in experiments 3 and 4 when they had to move in heavy smoke. This may suggest, that lack of visibility is the most probable reason for fear and discomfort rather than an unexpected situation and a sudden need for evacuation.

In their free observations participants, i.a. complain about low visibility in the main tunnel (8 times, mostly after Experiment 3). They wrote i.a.: *frightening feeling of lack of visibility*, *it was hard not to fall*, *I had to follow my intuition*. Interestingly, the same number of comments (8) state feeling fear and calmness during the evacuation.

**Fig 14:**
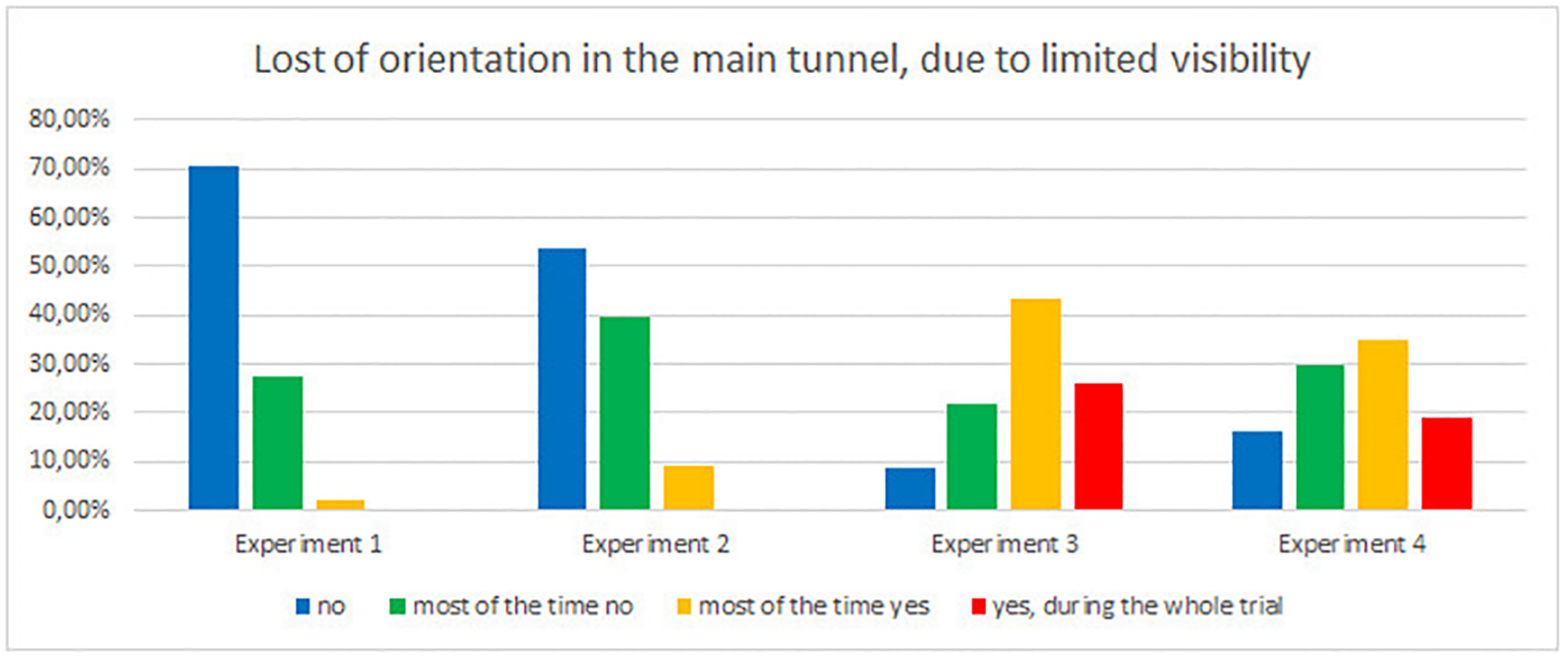
Problems with orientation in the main tunnel, due to limited visibility.

Other question investigate, if participants lose orientation during evacuation in the main tunnel (Fig 14). During experiment 1 pedestrians did not lose orientation in the tunnel due to smoke at all (70%) or most of the time (28%), for experiments 2 it is 54% and 40% respectively. On the other hand, during experiment 3, 43% of evacuees claim that they did lose orientation most of the time, or during the whole experiment (26%). Results for experiments 4 are similar, evacuees lost orientation only slightly less often.

**Fig 15:**
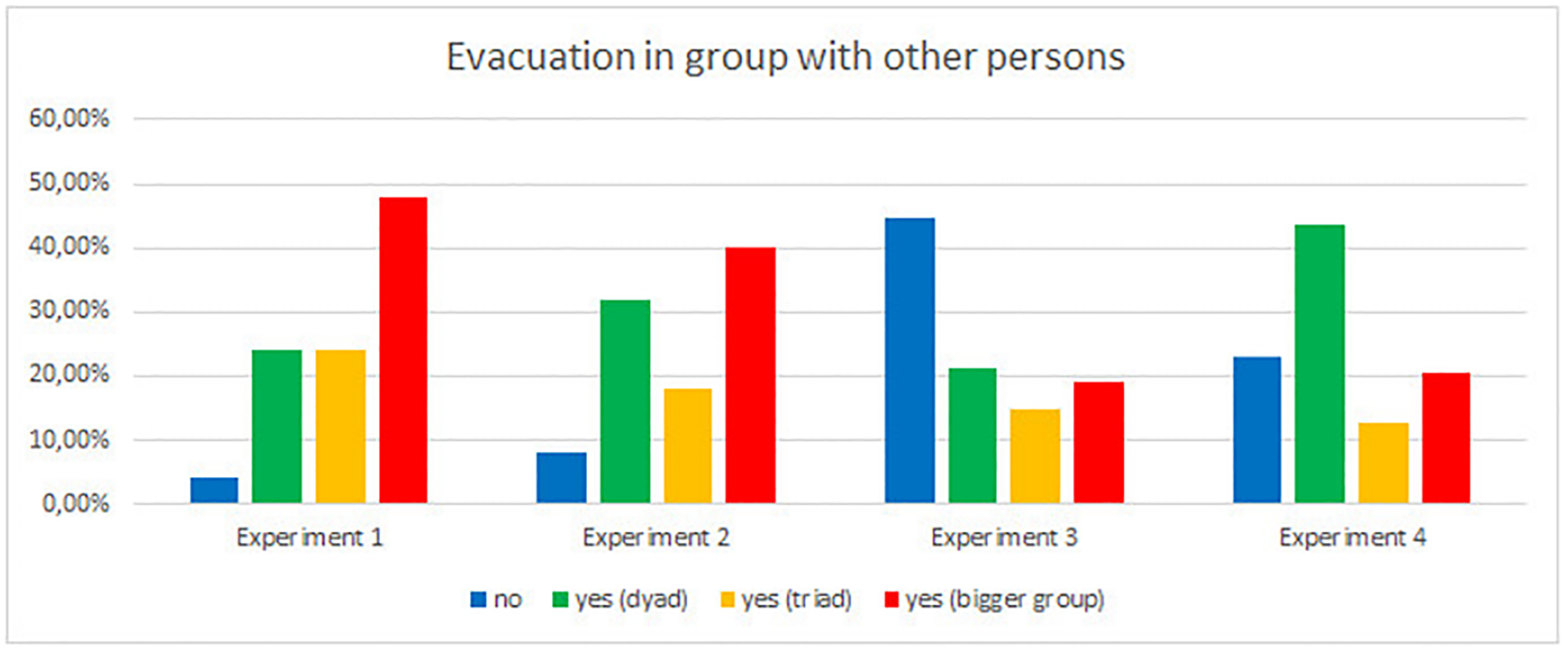
Grouping behavior during consecutive experiments.

Finally, we asked participants if they evacuated in groups (Fig 15). During the first experiment, almost half of the students pointed out that they evacuated in bigger groups (48%), while same number of students walked in dyads and triads (both 24%). It should be stressed that only 4% walked alone. In the second experiment we observed similar results, with some shift form triads and bigger groups to dyads. Grouping behavior changed completely in experiment 3 (competitive, low visibility). Here almost half of participants evacuate alone (45%). Finally, in experiment 4 (new place, low visibility) evacuees usually pointed out keeping in pairs (44%). These results clearly show the impact of external conditions on grouping behavior.

## 5 Discussion

Security issues in tunnels are the subject of extensive research, however real evacuation experiments from smoke filled tunnels are quite rare [10–12, 23, 31]. Previous studies focus mostly on: drivers making the decision to starting evacuating [11], walking speed in smoke [4, 12, 31], marking of evacuation exits [11, 12] and single person moving behavior in dense smoke [10, 12, 23]. In this research we analyze tunnel evacuation in the context of group behaviors and interactions between evacuees. According to our knowledge, this is the first tunnel evacuation experiment reported in a journal, that considers evacuation from a bus including the impact of smoke and grouping behaviors.

We observed the decision making of pedestrians in different densities of artificial smoke. During experiments: 0 (no smoke), 1 (light smoke *Cs* = 0.1-0.2 *m*^−1^) and 2 (moderate smoke *Cs* = 0.4—0.5 *m*^−1^), when visibility was good we observed that only the first group of passengers leaving the bus in the tunnel made a decision about escape direction. While during experiments 3 and 4 with heavy smokiness *Cs* = 0.8—0.9 *m*^−1^ and *Cs* = 1.0—1.1 *m*^−1^, respectively, each small group has to make a decision independently. Formation of such small groups is visible at check-points (Fig 5a and 5d), additionally participants reported in questionnaires and during informal surveys the feeling of uncertainty or fear, when exposed to increasingly dense smoke.

The grouping behavior of pedestrians is an important issue in our experiments. In heavy smoke, grouping behavior depends on evacuees’ density. For low crowd density, participants tend to keep in small groups (Fig 5b and 5d), while in higher crowd densities (experiments 3 and 4) *river like, double-line patterns*, patterns characteristic for bi-directional movement in a high density crowd in normal conditions can been seen. Such a pattern is seen in Fig 5c).

During experiments the appearance of a learning effect can also be observed. Pre-movement time, namely the time between an alarm signal and initialization of evacuation on the bus becomes shorter in consecutive experiments with the same participants. The time was reduced from 37*s* in experiment 1 down to 5*s* and respectively 3*s* in experiments 3 and 4. Analogically, evacuation time from the main tunnel, namely from the bus door checkpoint and cross-passage checkpoint, became increasingly shorter in consecutive experiments (48*s* in experiment 1 vs. 16 − 18*s* in 2, 3 and 4).

In experiment 3, with competitive conditions (with the task for participants to achieve the shortest possible personal evacuation time) we did not observe significant differences in evacuation times between the bus-door checkpoint and the cross-passage checkpoint (smoke filled area) against data from experiments 1 and 2 without the competitive conditions. While in the evacuation tube, an area without smoke the evacuation time between cross-passage checkpoint and evacuation tunnel checkpoint in experiment 3 was shorter. Outcomes from our questionnaire show that lack of visibility increases the feeling of fear more than participation in a new, unexpected situation, but with relatively good visibility (see Fig 13).

We show that beside smoke level, important factors which influence evacuees’ movement speed are their attitude (motivation) and familiarity with the situation (previous experiences and knowledge). Analysis of actual movement speed against desired speed are provided in order to properly calibrate numerical models for such events. A similar analysis of the dependency between movement speed in an area with and without smoke, was recently published by Ronchi et al. [9]. Both results seem to agree with the hypothesis stated in section 4.5. One of the interesting directions for further research is a detailed investigation on the relation between desired and actual speed in an area with smoke. It can be achieved by other experiments as well as a cross analysis of results from previous experiments [8, 9, 12, 23, 25].

In the current research the influence of obstacles like other cars was out of the scope (for research objectives see sec. 1). The presence of other vehicles, especially in smoke conditions would affect the evacuation process [11]. We notice that, as future work, more experiments with obstacles such as stopped cars should be carried out.

Further work are also required to fully understand the decision making process made by evacuees during a tunnel fire, both in case of decision on evacuation start and path selection.

## Supporting information

S1 Appendix Detailed information about an experiment. Tunnel architecture, detailed data of participants and the exact evacuation instructions given to participants after experiment 1.
(TEX)

S2 Appendix Survey for participants original version in Polish. (PDF)

S3 Appendix Survey for participants translated to English. (PDF)

S1 Data Evacuation times at chechpoints. (XLSX)

S1 Movie Infrared camera record from experiment 2. (MP4)

S2 Movie Infrared camera record from experiment 3. (MP4)

S3 Movie Infrared camera record from experiment 4. (MP4)
