# Supplementary material for: Pedestrian behavior during evacuation from road tunnel in smoke condition—Empirical results
Source: PLoS One. 2018 Aug 29;13(8):e0201732. doi: 10.1371/journal.pone.0201732 (PMC6114522; doi:10.1371/journal.pone.0201732)
Supplement: S2 Appendix — (PDF) [file pone.0201732.s002.pdf]

Ankieta dla uczestników eksperymentów w tunelu Emilia w Lalikach:

Dane ogólne:

Nr ID (numer naklejki/plastronu):	
Wiek:	
Płeć:	
Rok studiów i kierunek - specjalność:	
Wzrost:	
Waga *(w przybliżeniu – do oszacowania parametrów ruchu):*	
Szerokość ramion:	

1. Czy uczestniczyłeś kiedykolwiek wcześniej w ćwiczebnej bądź rzeczywistej ewakuacji tunelu?
   a. TAK
   b. NIE
2. Czy uczestniczyłeś kiedyś w jakiejkolwiek ewakuacji?
   a. TAK
   b. NIE
3. Czy zetknąłeś się kiedykolwiek z poruszaniem się/ewakuacją w warunkach zadymienia?
   a. TAK
   b. NIE
4. Czy znasz szczegółowe zasady zachowania w przypadku wybuchu pożaru w tunelu drogowym ?
   a. TAK
   b. NIE
   c. częściowo

Nr ID (numer naklejki/plastronu):	

1. **Zaznacz maksymalnie <u>dwie</u> podstawowe przesłanki w podjęciu decyzji o rozpoczęciu ewakuacji:**
   A - zatrzymanie autokaru, B - alarm pożarowy w tunelu, C - dym w tunelu, D - zachowanie innych osób, E - inne (wymień): ....
2. **Zaznacz maksymalnie <u>dwie</u> podstawowe przesłanki w podejmowaniu decyzji o wyborze drogi podczas ewakuacji:**
   A - naśladowanie innych, B- kierowanie się znakami ewakuacyjnymi, C - komunikaty głosowe, D - znajomość procedur ewakuacji, E – intuicja, F - decyzje na podstawie wcześniejszych doświadczeń, H - inne (wymień) ……………………………………………..
3. **Czy odczuwałeś strach lub niepewność podczas ewakuacji**
   A – nie, B – przez większość czasu nie, C – przez większość czasu tak, D – tak, podczas całej próby
4. **Czy zaobserwowałeś spadek widoczności na Twojej drodze poruszania ?**
   A *– nie, B – przez większość czasu nie, C – przez większość czasu tak, D – tak, przez cały czas*
5. <u>(Tylko w przypadku odpowiedzi B, C lub D w pytaniu 4)</u> **Czy w tunelu głównym traciłeś orientację w związku ze spadkiem widoczności?**
   A – nie, B – przez większość czasu nie, C – przez większość czasu tak, D – tak podczas całej próby
6. **Czy w trakcie ewakuacji trzymałeś się w jakiejś podgrupie z innymi osobami:**
   A – nie, B – tak (dwuosobowa), C – tak (trzyosobowa) , D – tak (więcej niż 3 osoby)

7. **Oceń swój stopień aktywności podczas próby**
   A – pełna aktywność, B – częściowa aktywność, C- niewielka aktywność, D – zupełna senność
8. **Oceń swój stopień zaangażowania/zainteresowania tą próbą**
   A – pełne zainteresowanie, B – częściowe zainteresowanie, C – niewielkie zainteresowanie, D – brak zainteresowania
9. **Oceń swoje samopoczucie podczas próby**
   A – bardzo dobre, B - dobre, C - średnie, D – złe
10. **Oceń swoje podejmowanie decyzji podczas próby ewakuacyjnej**
    A - pełne zdecydowanie, B - przez większość czasu zdecydowanie, C - małe zdecydowanie, D - wahanie, brak zdecydowania

11. **Oceń słyszalność komunikatów nadawanych przez system audio w tunelu:**
    A - rewelacyjna słyszalność, B - bardzo dobra słyszalność, C- dobra słyszalność, D - dostateczna słyszalność, E - bardzo słaba słyszalność, F - brak słyszalności
12. **Oceń oznakowanie dróg ewakuacyjnych:**
    A - rewelacyjne oznakowanie, B - bardzo dobre oznakowanie, C - dobre oznakowanie, D - dostateczne oznakowanie, E - bardzo słabe oznakowanie, F - niezauważalne oznakowanie
13. **Czy doświadczenia z poprzedniego eksperymentu były pomocne podczas Twojej ewakuacji:**
    A – w pełni pomocne, B - w większości pomocne, C - częściowo pomocne, D – niepomocne
14. **Czy instrukcja zachowania w przypadku wybuchu pożaru w tunelu drogowym była pomocna podczas Twojej ewakuacji:**
    A – w pełni pomocna, B - w większości pomocna, C -częściowo pomocna, D – niepomocna
15. **Opisz swoje obserwacje/wrażenia po przeprowadzonej próbie:**
