# Supplementary material for: Pedestrian behavior during evacuation from road tunnel in smoke condition—Empirical results
Source: PLoS One. 2018 Aug 29;13(8):e0201732. doi: 10.1371/journal.pone.0201732 (PMC6114522; doi:10.1371/journal.pone.0201732)
Supplement: S3 Appendix — (PDF) [file pone.0201732.s003.pdf]

SURVEY FOR PARTICIPANTS OF EXPERIMENT IN EMILIA TUNNEL IN LALIKI

General data:

Id number:	
Age:	
Gender:	
Year and discipline of studies:	
Height:	
Weight *(approximated):*	
Shoulders width:	

1. Have you ever participated in a trial or real tunnel evacuation?

a. YES b. NO

2. Have you ever participated in any evacuation?

a. YES b. NO

3. **Have you ever tried to move/evacuate in smoke conditions ?**

a. YES b. NO

4. **Do you know the exact rules of behavior in the case of fire in a road tunnel?**

a. YES b. NO c. partially

ID numer:	

1. **Choose one or two basic decision premises to start evacuation:**
   A - bus stoppage *B – fire drill* *C – smoke in the tunnel* *D – other people behavior*
   E – other :...

2. **Choose one or two basic decision premises to choose the path during evacuation:**
   A – mimicking other people *B- evacuation signs* *C – voice alarm messages*
   D – intuition, *D - knowledge of evacuation procedures* *F – earlier experiences*
   H - other ....

3. **Were you afraid or uncertain during the evacuation?**
   A – no *B – most of the time no* *C – most of the time yes* *D – yes, during whole trial*

4. **Did you observe a decrease in visibility on your path?**
   A – no *B – most of the time no* *C – most of the time yes* *D – yes, during whole trial*

5. (Only if you have choosen B, C or D in question 4)
   Did you lose orientation in the main tunnel, due to limited visibility?
   A – no *B – most of the time no* *C – most of the time yes* *D – yes, during whole trial*

6. **Did you evacuate in a group with other people ?**
   A – no *B –* yes (dyad), *C –* yes (triad), *D –* yes (bigger group)

7. **Evaluate your activity level during this trial.**
   A – full activity *B – partial activity* *C – low actvity* *D – lack of actvity*

8. **Evaluate your involvement level during this trial.**
   A – full involvement *B – partial involvement* *C – low involvement,* *D – lack of involvement*

9. **Evaluate your comfort level during this trial.**
   A – very good *B - good* *C - average* *D – bad*

10. **Evaluate your decisiveness during this trial.**
    A – full decisiveness, *B - decisiveness by most of the time,* *C - average,* *D – lack of decisiveness*

11. **Evaluate the transmission of information of the voice alarm system:**
    A – excellent audibility B – very good audibility C- good audibility
    D – passable audibility E – very weak audibility F – no audibility

12. **Evaluate escape route markings:**
    A – excellent marking B – very good marking C- good marking
    D – passable marking E – very week marking F – unnoticeable marking

13. **Were your experiences from previous trials helpful during this evacuation:**
    A – fully helpful, *B – mostly helpful,* *C – partlially helpful,* *D – not helpful at all*

14. **Were the fire and evacuation instructions for the road tunnel helpful during this evacuation:**
    A – fully helpful, *B – mostly helpful,* *C – partlially helpful,* *D – not helpful at all*

15. **Describie your observations and impressions from this trial.**
